# Innate immune responses following Kawasaki disease and toxic shock syndrome

**DOI:** 10.1371/journal.pone.0191830

**Published:** 2018-02-15

**Authors:** Katherine Y. H. Chen, Nicole Messina, Susie Germano, Rhian Bonnici, Bridget Freyne, Michael Cheung, Greta Goldsmith, Tobias R. Kollmann, Michael Levin, David Burgner, Nigel Curtis

**Affiliations:** 1 Murdoch Children’s Research Institute, The Royal Children’s Hospital, Melbourne, Vic, Australia; 2 Department of Paediatrics, The University of Melbourne, Melbourne, Vic, Australia; 3 Infectious Diseases Unit and Department of General Medicine, The Royal Children’s Hospital, Melbourne, Vic, Australia; 4 Heart Research Group, Murdoch Children’s Research Institute and Department of Cardiology, The Royal Children’s Hospital, Melbourne, Vic, Australia; 5 Division of Infectious Diseases, Department of Pediatrics, University of British Columbia, Vancouver, BC, Canada; 6 Paediatric Infectious diseases group, Division of Medicine, Imperial College London, London, United Kingodm; 7 Department of Paediatrics, Monash University, Melbourne, Vic, Australia; Katholieke Universiteit Leuven Rega Institute for Medical Research, BELGIUM

## Abstract

The pathogenesis of Kawasaki disease (KD) remains unknown and there is accumulating evidence for the importance of the innate immune system in initiating and mediating the host inflammatory response. We compared innate immune responses in KD and toxic shock syndrome (TSS) participants more than two years after their acute illness with control participants to investigate differences in their immune phenotype. Toxic shock syndrome shares many clinical features with KD; by including both disease groups we endeavoured to explore changes in innate immune responses following acute inflammatory illnesses more broadly. We measured the *in vitro* production of interferon (IFN)-γ, tumour necrosis factor (TNF)-α, interleukin (IL)-1β, IL-6, IL-1 receptor antagonist (IL-1ra), and IL-10 following whole blood stimulation with toll-like receptor and inflammasome ligands in 52 KD, 20 TSS, and 53 control participants in a case-control study. Analyses were adjusted for age, sex, and unstimulated cytokine concentrations. Compared to controls, KD participants have reduced IL-1ra production in response to stimulation with double stranded RNA (geometric mean ratio (GMR) 0.37, 95% CI 0.15, 0.89, p = 0.03) and increased IL-6 production in response to incubation with Lyovec™ (GMR 5.48, 95% CI 1.77, 16.98, p = 0.004). Compared to controls, TSS participants have increased IFN-γ production in response to peptidoglycan (GMR 4.07, 95% CI 1.82, 9.11, p = 0.001), increased IL-1β production to lipopolysaccharide (GMR 1.64, 95% CI 1.13, 2.38, p = 0.01) and peptidoglycan (GMR 1.61, 95% CI 1.11, 2.33, p = 0.01), and increased IL-6 production to peptidoglycan (GMR 1.45, 95% CI 1.10, 1.92, p = 0.01). Years following the acute illness, individuals with previous KD or TSS exhibit a pro-inflammatory innate immune phenotype suggesting a possible underlying immunological susceptibility or innate immune memory.

## Introduction

Kawasaki disease (KD) is a systemic vasculitis most commonly affecting pre-school children characterised by fever, rash, conjunctivitis, lymphadenopathy, mucosal and peripheral changes in the hands and feet.[1] Its pathogenesis remains unknown, leading to many uncertainties in the diagnosis and treatment of the disease. The main complication of KD is the development of coronary artery aneurysms.[2] It is the leading cause of acquired heart disease in children living in industrialised countries.

Although it is widely believed that KD is caused by an infectious trigger, there is growing evidence that genetic factors play a role in determining susceptibility and severity of the disease.[2] Evidence for host susceptibility comes from epidemiological data. The incidence of KD remains highest in children of Asian ethnicity with Japan reporting over 10 times the incidence rate compared to United States for children under five years of age.[3, 4] This increased incidence is evident in children of Asian migrants living in low incidence countries, and in first degree relatives of KD patients. [5–7] Furthermore, genome-wide association and candidate gene studies have identified a number of associations with genes involved in the regulation of the inflammatory process, including *ITPKC* and *ORAI1*, which regulate intracellular calcium-signalling pathways.[8–11] These genetic associations support the widely accepted hypothesis that KD occurs in genetically susceptible individuals in whom microbial pathogens or environmental agents trigger an aberrant inflammatory response.[2]

There is increasing interest in the role of the innate immune system in the generation of this aberrant inflammatory response in KD.[12] During the acute illness, peripheral neutrophils and monocytes increase and infiltrate affected arterial walls, leading to acute self-limited necrotising arteritis.[13–16] Acutely, KD patients have increased toll-like receptor (TLR) mRNA levels and upregulation of genes in the IL-1 pathway.[17, 18] Furthermore, human and murine data implicate a critical role for nucleotide-binding domain and leucine-rich repeat pyrin domain contain 3 (NLRP3) inflammasome activation in acute KD, and in the development of coronary artery aneurysms. Modulation of IL-1 is therefore a potential therapeutic target.[19–22]

Little is known about the immune responses to microbial pathogens years after an acute episode of KD. We hypothesised that an abnormal inflammatory phenotype in KD patients, measured years after the acute illness, might provide clues to the nature of the aberrant immune response in acute KD and reveal targets for anti-inflammatory treatment.

Toxic shock syndrome (TSS), a prototypical illness caused by bacterial superantigens, shares many clinical and immunological features with KD.[23] In particular, KD shock syndrome, characterised by myocardial dysfunction, coronary artery involvement and resistance to standard therapy, is the most difficult to clinically differentiate from TSS.[24] There is also emerging interest in the role of the innate immune system in initiating and mediating the host inflammatory response in the development of sepsis.[25] Similar to KD, there is likely an underlying host immunological susceptibility as not all individuals exposed to the causative exotoxin develop TSS.[26, 27] By including both diseases, we were able to study changes in innate immune responses following acute inflammatory illnesses more broadly.

We compared the innate immune responses in participants with previous KD or TSS with control participants at least two years after their acute illness. Our aim was to define an innate immune phenotype that might suggest either an underlying immunological susceptibility and/or evidence of innate immune memory.

## Material and methods

### Participants and enrolment

Participants between six and 30 years of age who had KD or TSS at least two years previously were recruited from The Royal Children’s Hospital (RCH) and Monash Medical Centre (MMC), Melbourne, Australia. A research letter of invitation was sent out to families meeting the inclusion criteria following a medical record search.

The KD group included participants with a history of acute KD that fulfilled either the American Heart Association diagnostic criteria for KD,[28] and/or had abnormal coronary artery dimensions that met the Japanese Ministry of Health Criteria for abnormal dilatation[29] on an echocardiogram performed within two months of disease onset. Height is not routinely measured on children acutely hospitalised in Australia and so it was not possible to generate coronary artery z-scores, which are based on calculated body surface area.[30] The KD group were therefore defined by widely accepted and robust criteria.

The TSS group included patients who fulfilled the Centers for Disease Control and Prevention case definition for either probable or definite TSS.[31] Control participants were unrelated healthy individuals of similar age and sex recruited from The Royal Children’s Hospital Melbourne clinics, or unrelated friends of KD and TSS participants. Exclusion criteria for all groups were treatment for hypertension and/or dyslipidaemia, or chronic auto-inflammatory conditions. Assessments were deferred for at least four weeks following an acute febrile illness. The study was approved by the human research ethics committee at RCH (Approval No 31090) and Monash (11293X) and written informed consent was obtained from the parents or adult participants at enrolment. Participants attended the Murdoch Children’s Research Institute (Melbourne, Australia) on a single occasion between September 2013 and December 2015 during which anthropometric measurements (BC 418, Tanita, Tokyo, Japan), as well as demographic and clinical details were obtained. Venous blood was collected into lipopolysaccharide (LPS)-free sodium heparin, serum gel, and ethylenediamine tetraacetic acid (EDTA) tubes (Sarstedt, Germany). High sensitivity C-reactive protein (hsCRP) (Abbott Architect, IL, USA) was measured from the serum gel tube according to manufacturer’s protocol.

### *In vitro* stimulation assays

To standardise assays, all TLR and inflammasome ligands were pre-prepared in robotically-filled 8-well strips within 96-well plates (Corning, USA) at the University of British Columbia, Vancouver, as previously described.[32] The wells in the TLR strips contained the following ligands: 3.5 μg/mL R848 (Resiquimod) (TLR 7/8 ligand, InvivoGen, USA); 100 ng/ml LPS (TLR 4, InvivoGen); 10 μg/ml peptidoglycan (PGN) (nucleotide-binding oligomerization domain (NOD) 1/2, InvivoGen); 100 μg/ml polyinosinic-polycytidylic acid (Poly I:C) (TLR 3, Amersham Biosciences, UK); 20 μg/ml cyclic guanosine monophosphate-adenosine monophosphate (cGAMP) (Stimulator of the interferon gamma gene (STING) ligand, InvivoGen) with Lyovec™ (InvivoGen); 2 ng/mL double stranded RNA (dsRNA) (Retinoic acid-inducible gene (RIG) 1, InvivoGen) with Lyovec™; and RPMI 1640 (Gibco, USA) and Lyovec™ (unstimulated). The NLRP3 inflammasome strip-wells contained 100 μg/ml Zymosan (Sigma-Aldrich), 100 ng/ml LPS (InvivoGen), 5 mM/mL adenosine triphosphate (ATP) (InvivoGen), and RPMI (unstimulated).

One hour after collection, heparinised fresh blood was diluted with RPMI to a final 1:1 dilution by adding 180 μl of the diluted blood to each well of the pre-prepared thawed and centrifuged strips containing 20 μl of the specific ligand per well. Samples stimulated with NLRP3 inflammasome ligands were incubated for two hours, while samples containing TLR ligands were incubated for 24 hours at 37°C in 5% CO_2_. Pre-prepared ATP was thawed and added to the Zymosan and LPS wells on the inflammasome strips 30 minutes before harvesting.[33] Following incubation, the strip-wells were centrifuged at 600 *g* for 5 minutes and the supernatants stored in microtubes (VWR, USA) at -80°C for future batched analysis.

### Cytokine analysis

The choice of cytokines measured was based on innate immune pathways for each ligand. Cytokine concentrations from TLR ligand-stimulated supernatants were measured using an x-MAP immunobead-based multiplexed assay (Bio-Plex Pro Human Cytokine Group 1 Assay, Bio-Rad, USA) according to manufacturer’s protocol. Samples from KD, TSS, and control participants were scrambled across plates. To ensure that cytokine concentrations of stimulated samples were within the detectable range, samples were diluted 1:4 for the measurement of interferon (IFN)-γ, tumour necrosis factor (TNF)-α, and interleukin (IL)-10 and 1:100 for the measurement of IL-1β, IL-1 receptor antagonist (IL-1ra), and IL-6. In preliminary experiments, IFN-α and IFN-β concentrations from samples diluted 1:4 were below the detection limit for the majority of samples in 8 out of 10 participants (3 KD, 3 TSS and 4 control participants) despite measurable concentrations for other cytokines. These two cytokines were therefore not measured for the remaining participants.

Interleukin-1β levels were measured in the NLRP3 inflammasome ligand-stimulated supernatant using an enzyme-linked immunosorbent assay (Mabtech, Australia) according to the manufacturer’s protocol using samples diluted 1:50.

### Statistical analysis

Statistical analysis was done using Stata 13.0 (Stata Corporation, Texas). Cytokine concentrations below the detection limit were designated a value half the lowest detection limit for that specific cytokine. Outlier participants were defined as those having two or more unstimulated cytokine concentrations greater than two standard deviations above the mean value of the other participants and were excluded.

Cytokine concentrations were analysed as continuous variables and were log transformed to achieve a normal distribution. Geometric mean (95% CI) is the mean of the logarithmic values retransformed back to picogram/ml (pg/ml) using the exponential function. Univariate comparisons of cytokine concentrations were done using 2-tailed *t*-test.

Multi-variable linear regression was used to quantify the differences between groups adjusting for unstimulated cytokine concentrations (either with RPMI or Lyovec™), age and sex with results expressed as geometric mean ratio (GMR).

Cytokine concentrations from the inflammasome strip-wells could not be transformed into a normal distribution and, therefore, comparisons between participant groups were done using the Kruskal-Wallis test after nil correction.

## Results

In total, 60 KD, 22 TSS, and 60 control participants were recruited. Ninety two eligible KD participants were approached and 60 were recruited. The number of participants with coronary artery abnormalities in the recruited (35/60 (58.3%)) and the declined group (18/32 (56.3%) were similar.

Two participants (one TSS and one control) were considered outliers and another two KD participants were excluded on the basis that they had a febrile illness on the day of the assessment. A further 13 participants (6 KD, 1 TSS, 6 controls) were excluded due to insufficient blood to complete stimulations assays. There were, therefore, 52 KD, 20 TSS, and 53 control participants included in the final analysis. There were no differences in age, sex, anthropometry, pubertal status and hsCRP levels between the groups (Table 1).

**Table 1:** Participant characteristics.

	Kawasaki disease (n = 52)	Toxic shock syndrome (n = 20)	Control (n = 53)
*Age (years)*	15.5 ± 5.8	15.6 ± 5.6	14.9 ± 6.1
Sex (male (%))	30 (57.7%)	12 (57.1%)	26 (48.2%)
Height (cm)	158.7 ± 19.2	162.2 ± 19.5	155.3 ± 17.1
Weight (kg)	54.0 ± 18.1	59.7 ± 23.3	51.1 ± 18.3
Post-pubertal^a^ (n %)	24 (46.2%)	10 (47.6%)	20 (37.0%)
Age at acute illness (years)	3.3 ± 3.0	8.7 ± 4.5	N/A
Time since acute illness (years)	12.2 ± 5.8	5.9 ± 3.1	N/A
High sensitive CRP (mg/L)	0.6 (0.3–1.4)	0.5 (0.3–0.7)	0.4 (0.2–0.8)
Ethnicity (n%)			
Caucasians	40 (69.8%)		37 (73.3%)
Asians	9 (17.0%)		9 (17.1%)
Others	7 (13.2%)		3 (9.6%)

Mean ± SD for normally distributed data, median (interquartile range) for skewed data

^a^self-reported Tanner stage 5

CRP = C-reactive protein

Thirty nine (75%) participants met the American Heart Association complete diagnostic criteria for KD. Thirteen participants (25%) met the incomplete diagnostic criteria and fulfilled the Japanese Ministry of Health echocardiogram criteria for coronary artery abnormality. There were 20 (38.5%) participants with no history of coronary artery abnormalities, 18 (34.6%) with resolved dilatation or coronary artery aneurysms, and 14 (26.9%) with persistent coronary artery aneurysms. Ten (19.2%) were resistant to the first dose of intravenous immunoglobulin. All received a second dose of intravenous immunoglobulin and two (3.8%) received steroids in addition.

Of the 20 TSS participants in the final analysis nine (45%) met the definite TSS diagnostic criteria and 11 (55%) met the probable TSS criteria.

### Toll-like receptor ligand stimulation

**Fig 1:**
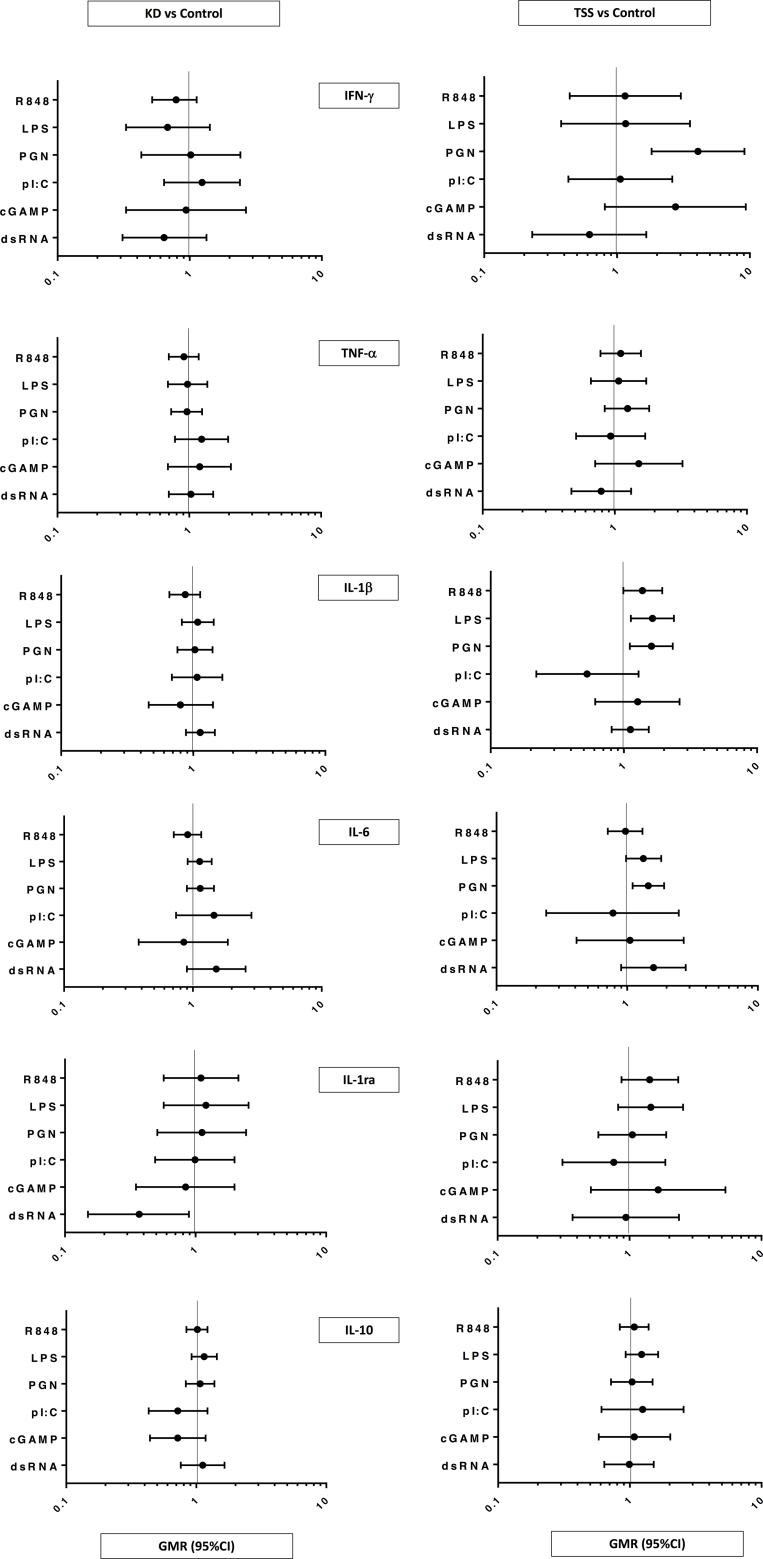
Geometric mean ratio (GMR) and 95% CI of *in vitro* toll-like receptor induced cytokine concentrations in 52 Kawasaki disease (KD), 20 toxic shock syndrome (TSS) and 53 control participants adjusted for age, sex and unstimulated cytokine concentrations. Abbreviations: IFN, interferon; TNF, tumour necrosis factor; IL, interleukin; IL-1ra, receptor antagonist; R848, Resiquimod; LPS, lipopolysaccharide; PGN, peptidoglycan; pI:C, polyinosinic-polycytidylic acid; cGAMP, cyclic guanosine monophosphate-adenosine monophosphate; dsRNA, double stranded RNA.

Compared to controls, KD participants had reduced IL-1ra production in response to dsRNA (GMR 0.37, 95% CI 0.15, 0.89, p = 0.03). There were no differences in pro-inflammatory cytokine production in response to TLR ligands between KD and control participants (Fig 1).

Compared to controls, TSS participants had increased IFN-γ production in response to PGN stimulation (GMR 4.07, 95% CI 1.82, 9.11, p = 0.001), increased IL-1β production to LPS (GMR 1.64, 95% CI 1.13, 2.38, p = 0.01) and PGN (GMR 1.61, 95% CI 1.11, 2.33, p = 0.01), and increased IL-6 production to PGN (GMR 1.45, 95% CI 1.10, 1.92, p = 0.01) after adjusting for unstimulated cytokine concentrations, age and sex. There were no differences in anti-inflammatory cytokine production between TSS and control participants (Fig 1).

Unadjusted geometric means of cytokines following TLR ligand stimulations are shown in S1 Table. The direction and magnitude of change were similar to the adjusted analysis with one exception. In the unadjusted analysis, there was an increased production of IL-6 in response to dsRNA stimulation in KD participants which was not statistically significant after adjusting for unstimulated cytokine concentrations, age and sex.

**Fig 2:**
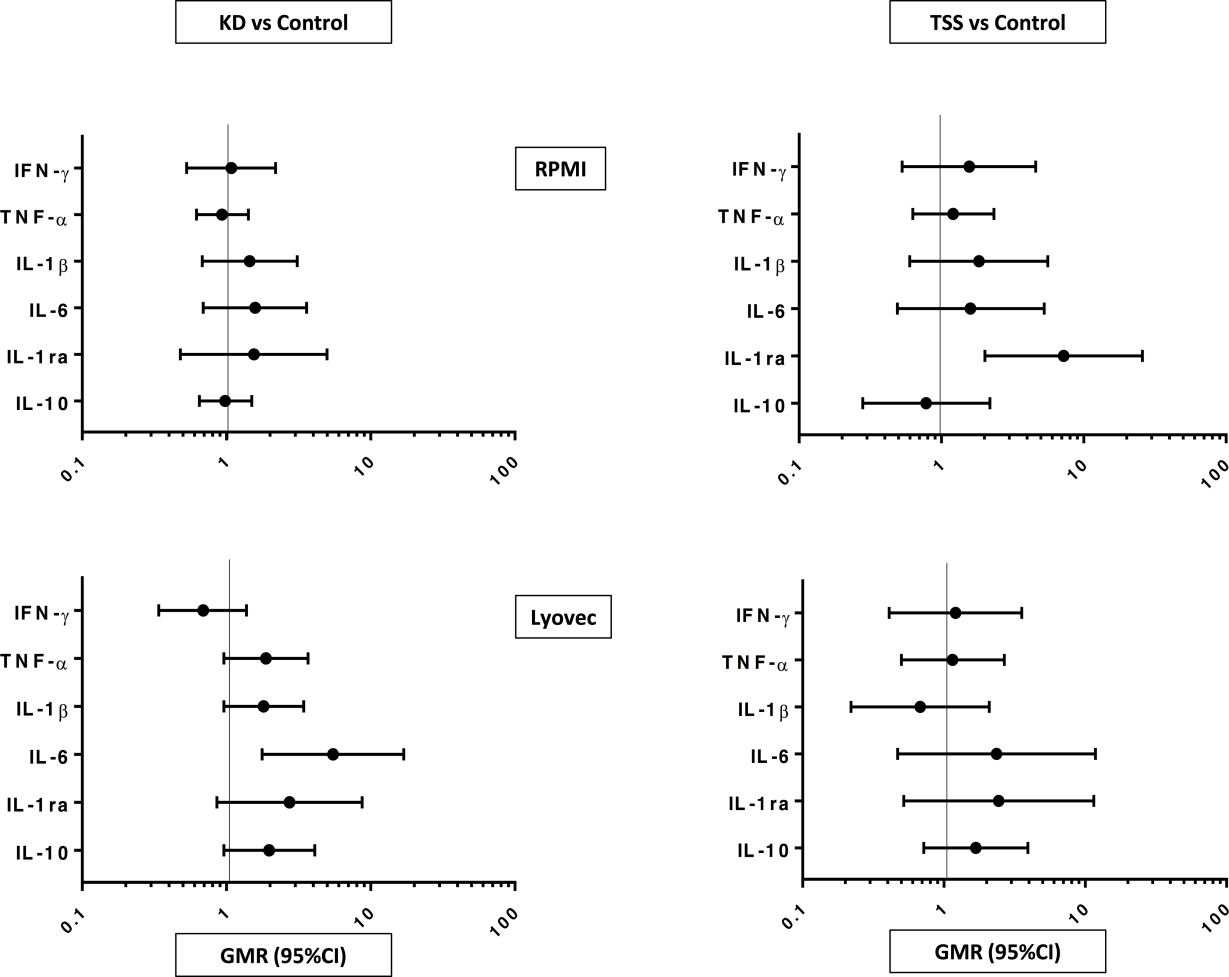
Geometric mean ratio (GMR) and 95% CI of *in vitro* cytokine concentrations in the unstimulated RPMI and Lyovec™ wells of 52 Kawasaki disease (KD), 20 toxic shock syndrome (TSS) and 53 control participants. Abbreviations: IFN, interferon; TNF, tumour necrosis factor; IL, interleukin; IL-1ra, receptor antagonist.

Cytokine responses in the ‘unstimulated’ wells incubated with Lyovec™ were consistently higher than those observed in the wells incubated with RPMI with the notable exception of IFN-γ (S1 Table). Compared with the controls, KD participants had a higher geometric mean IL-6 concentration in samples incubated with Lyovec™, and the TSS participant group had a higher geometric mean IL-1ra concentration in samples incubated with RPMI (S1 Table and Fig 2).

### Inflammasome ligand stimulation

**Fig 3:**
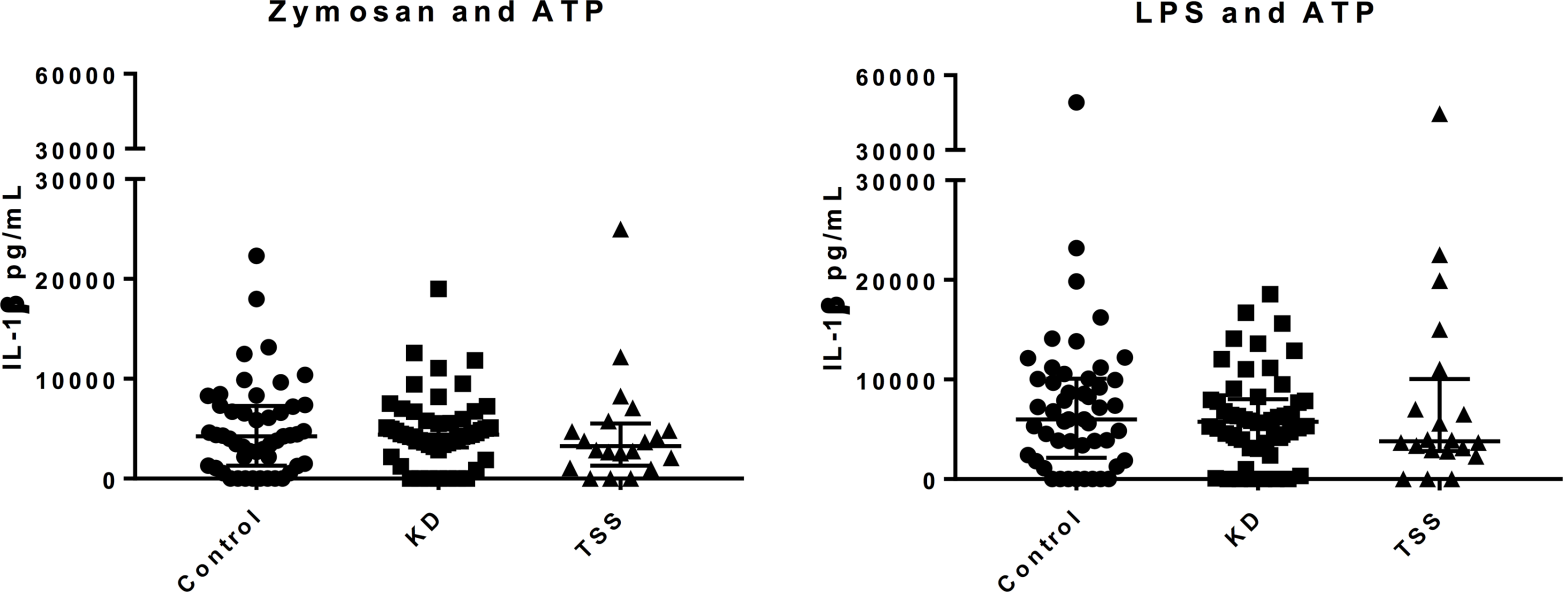
Scatter plot of interleukin (IL)-1β concentrations in 52 Kawasaki disease (KD), 20 toxic shock syndrome (TSS) and 53 control participants following *in vitro* stimulations of the nucleotide-binding domain and leucine-rich repeat pyrin domain containing 3 (NLRP3) inflammasome. The median and interquartile ranges are marked by the bars. Abbreviations: pg, picogram; ATP, adenosine triphosphate.

No differences were found in IL-1β production between KD, TSS, and control participants in response to Zymosan plus ATP (median (IQR): 4388.25 (3189.00, 5785.25) vs 3249.85 (1559.53, 5276.95) vs 4229.20 (1304.70, 7185.30) pg/ml, p = 0.6) and LPS plus ATP (5760.10 (3632.55, 7941.00) vs 3777.48 (2843.43, 9021.60) vs 5995.10 (2396.80, 10032.40) pg/ml, p = 0.7) stimulations of the NLRP3 inflammasome (Fig 3).

## Discussion

This is the first study to report the innate immune responses in individuals with previous KD and TSS years after the acute illness. The observed decreased IL-1ra production in KD participants with a prototypical viral ligand stimulation is consistent with data implicating the role of IL-1β in the pathogenesis of KD. Studies have shown elevated IL-1β in acute KD especially in treatment resistant individuals, increased transcript abundance for IL-1β related genes, and protection from coronary arteritis in IL-1βreceptor-deficient mice.[19, 20, 34] Randomised controlled trials using an IL-1β receptor antagonist and monoclonal antibody inhibition of IL-1β in KD are underway.[19–22] We found no differences in IL-1β production in KD participants following both TLR and inflammasome stimulation, which is consistent with studies in convalescent KD.[19] Inflammasome activation and resulting elevated levels of IL-1β are most likely transient acute inflammatory responses.

The observation that cytokine concentrations in samples incubated with Lyovec™ were higher than those incubated with RPMI suggests that this lyophilized cationic lipid-based transfection reagent stimulates cytokine responses. Cationic lipids such as Lyovec™ are able to activate pro-inflammatory pathways, in particular, intracellular calcium-signalling pathways.[35] These pathways have been implicated in the pathogenesis of KD.[9, 19] The finding that Lyovec™ was associated with a greater IL-6 response in the KD group compared to the control group may point to a genetically determined difference in the response to intracellular pattern recognition receptor stimulation by lipids from the cell wall of bacteria or fungi. This might suggest that KD patients have an underlying different inflammatory response involving these pathways.

This study does not provide evidence for either a conventional or superantigen as the initial trigger for KD as superantigens were not used as a ligand in our study. Superantigens will likely be neutralised with the use of whole blood required to activate the innate immune system in our study.

Toxic shock syndrome participants had an increased pro-inflammatory cytokine response to bacterial ligands, which is consistent with reported cytokine responses to TSS toxin (TSST)-1 *in vitro*.[36] Most cases of TSS are caused by exotoxins produced by *Staphylococcus aureus* and *Streptococcus pyogenes*, which act as ‘superantigens’ binding directly to the major histocompatibility complex class II molecule and T-cell receptor, bypassing the conventional antigen-binding sites.[23] Conceivably, similar to LPS and PGN ligand stimulation in our study, these bacteria would also stimulate the host innate immune system through activation of TLR 2 and NOD receptors[37]

The underlying immunological and/or genetic susceptibility determinants of TSS are poorly understood. The prevalence of antibody to TSST-1 in the population far outweighs the number of TSS cases.[26, 27] The observed increased pro-inflammatory cytokine response to TLR 2 and NOD receptor stimulation in TSS may be due to either an underlying genetic immunological susceptibility or an altered immune response secondary to the past TSS illness. The possibility that our results reflect an underlying genetic immunological susceptibility is supported by the finding of a strong genetic influence on *in vitro* LPS-induced cytokine responses in meningococcal disease in a study of first degree relatives.[38]

Of note, compared to control participants, IL-1β production following inflammasome stimulation was not increased in TSS participants despite increased production following TLR stimulation, which theoretically share the same pathway for IL-1 activation. It is possible that LPS and PGN may have stimulated the activation of caspase (which in turn activates IL-1) through other less well characterised pathways, such as RIG-I or IFN-γ inducible protein 16.[39] Furthermore, differences in methodologies between the TLR and NLRP3 inflammasome strip stimulation assays, especially in the incubation time, make direct comparison of results difficult.

The strengths of our study are the use of well-characterised KD and TSS participants, and rigorously standardised assays. Limitations of our study include its retrospective design and therefore inability to differentiate whether the innate immune response differences preceded KD or TSS (reflecting potential underlying immunosusceptibility) versus those attributable to the effect of diseases (innate immune memory). We are also unable to ascertain whether these innate immune responses persist into later life and were related to time following the acute illness. Furthermore, the purpose of this study was to explore whether there are changes in the innate immune response in two acute illnesses with overlapping clinical features, rather than to directly compare KD and TSS, moreover, the sample size is insufficient.

As this is an exploratory study, analyses have not been adjusted for multiple comparisons. The exploratory nature of this study necessarily required the inclusion of numerous cytokines measures to investigate multiple innate immune pathways. We did not adjust each measure by severity indicators (such as coronary artery status, peak inflammatory markers, duration of fever, and treatment resistance) as the small number of participants in this subset analysis would risk Type 1 and Type 2 errors. The impact of disease severity on innate immune responses would best be explored in future studies with sufficient sample size focusing on a small number of implicated cytokines.

There is increasing interest in the concept that innate immune cells also have the capacity to enhance their response following repeated stimulation by microbial pathogens through epigenetic programming.[40] Innate immune memory has important implications for understanding host response to pathogens and auto-inflammatory conditions. Hyper-inflammatory innate immune responses, particularly noted in TSS participants, may indicate innate immune memory and warrant further investigation.

In summary, years following the acute illness, individuals with previous KD and TSS have a decreased anti-inflammatory and increased pro-inflammatory response respectively to innate immune stimulation, suggesting a possible underlying immunological susceptibility or innate immune memory. Further genetic and immunological studies may unravel the mechanisms underlying these responses, which have implications for improved diagnostics and treatment.

## Supporting information

S1 Table Geometric mean (95% CI) of stimulated *in vitro* cytokine concentrations in pg/ml. (DOCX)

## Abbreviations

KD: Kawasaki disease
TSS: toxic shock syndrome
GMR: geometric mean ratio
IFN-γ: interferon-γ
TNF-α: tumour necrosis factor-α
IL-6: interleukin-6
IL-1ra: IL-1 receptor antagonist
ITPKC: inositol-trisphosphate-kinase C
NLRP3: nucleotide-binding domain and leucine-rich repeat pyrin domain contain 3
TLR: toll-like receptor
LPS: lipopolysaccharide
EDTA: ethylenediamine tetraacetic acid
hsCRP: high sensitivity C-reactive protein
PGN: peptidoglycan
NOD: nucleotide-binding oligomerization domain
RIG: retinoic acid-inducible gene
STING: Stimulator of the interferon gamma gene
Poly I:C: polyinosinic-polycytidylic acid
cGAMP: cyclic guanosine monophosphate-adenosine monophosphate
dsRNA: double stranded RNA
TSST-1: toxic shock syndrome toxin-1
