# Supplementary material for: Innate immune responses following Kawasaki disease and toxic shock syndrome
Source: PLoS One. 2018 Feb 15;13(2):e0191830. doi: 10.1371/journal.pone.0191830 (PMC5813928; doi:10.1371/journal.pone.0191830)
Supplement: S1 Table — (DOCX) [file pone.0191830.s001.docx]

# Supporting information

S1 table Geometric mean (95% CI) of stimulated in vitro cytokine concentrations in pg/ml

|  | **Proportion of observations within detection limit** | **Kawasaki disease**  **(n=52)** | **Toxic shock syndrome**  **(n=20)** | **Control**  **(n=53)** |
| --- | --- | --- | --- | --- |
| **IFN-γ** |  |  |  |  |
| RPMI | 54 (43%) | 3.45 (2.07, 5.75) | 5.05 (1.98, 12.85) | 3.45 (2.07, 5.75) |
| Lyovec™ | 58 (46%) | 3.07 (1.87, 5.05) | 5.15 (1.96, 13.55) | 4.42 (2.78, 7.04) |
| R848 | 126 (99%) | 953.96 (715.41, 1272.06) | 1626.22 (683.69, 3868.12) | 1242.51 (934.89, 1651.36) |
| LPS | 117 (92%) | 99.72 (56.47, 176.10) | 192.95 (71.71, 519.15) | 144.68 (89.56, 233.73) |
| PGN | 104 (82%) | 35.41 (18.97, 66.09) | 145.78 (86.59, 245.43) | 33.17 (18.77, 58.60) |
| pI:C | 122 (96%) | 231.40 (140.28, 381.70) | 203.78 (97.55, 425.66) | 177.77 (118.29, 267.17) |
| cGAMP | 97 (76%) | 30.51 (14.73, 63.19) | 92.34 (35.33, 241.35) | 33.44 (17.04, 65.64) |
| dsRNA | 67 (53%) | 4.27 (2.43, 7.52) | 5.20 (1.80, 14.96) | 7.75 (4.61, 13.01) |
| **TNF-α** |  |  |  |  |
| RPMI | 84 (66%) | 6.55 (4.77, 8.99) | 8.74 (4.94, 15.47) | 7.18 (5.49, 9.40) |
| Lyovec™ | 113 (89%) | 52.58 (32.67, 84.62) | 33.33 (16.57, 67.01) | 29.49 (18.35, 47.40) |
| R848 | 127 (100%) | 3167.97 (2571.42, 3902.92) | 4132.91 (2932.46, 5824.77) | 3544.67 (2936.53, 4278.77) |
| LPS | 127 (100%) | 2390.44 (1841.46, 3103.09) | 2828.84 (1861.21, 4299.52) | 2546.24 (1971.02, 3289.32) |
| PGN | 127 (100%) | 1418.68 (1123.56, 1791.32) | 1907.41 (1355.69, 2683.66) | 1495.30 (1221.37, 1830.68) |
| pI:C | 125 (98%) | 71.21 (47.64, 106.44) | 58.65 (32.83, 104.78) | 56.94 (44.67, 72.58) |
| cGAMP | 126 (99%) | 321.71 (213.38, 485.03) | 393.70 (191.75, 808.31) | 247.02 (174.18, 350.32) |
| dsRNA | 124 (98%) | 285.33 (215.35, 378.07) | 195.14 (126.50, 301.02) | 229.57 (163.77, 321.79) |
| **IL-1β** |  |  |  |  |
| RPMI | 86 (68%) | 16.05 (9.73, 26.49) | 20.15 (7.92, 51.27) | 10.97 (6.30, 19.10) |
| Lyovec™ | 110 (86%) | 80.67 (53.12, 122.48) | 31.06 (11.88, 81.23) | 46.92 (28.65, 76.86) |
| R848 | 125 (98%) | 1536.26 (1275.13, 1850.87) | 2435.39 (1844.92, 3214.83) | 1766.71 (1413.09, 2208.83) |
| LPS | 125 (98%) | 2603.72 (2119.75, 3198.18) | 3937.26 (2851.64, 5436.18) | 2454.13 (1997.20, 3015.60) |
| PGN | 125 (98%) | 821.78 (664.11, 1026.89) | 1281.54 (964.32, 1703.12) | 791.11 (622.75, 1004.99) |
| pI:C | 120 (94%) | 142.31 (99.43, 203.68) | 80.96 (33.15, 197.72) | 122.32 (96.12, 155.65) |
| cGAMP | 114 (90%) | 82.58 (52.31, 130.38) | 86.70 (36.18, 207.77) | 82.20 (55.79, 121.11) |
| dsRNA | 125 (98%) | 341.23 (281.41, 413.75) | 308.63 (240.11, 396.72) | 277.92 (233.14, 331.30) |
| **IL-6** |  |  |  |  |
| RPMI | 63 (50%) | 39.26 (22.18, 69.49) | 38.61 (14.11, 105.64) | 24.83 (13.99, 44.06) |
| Lyovec™ | 102 (80%) | 1829.24 (889.72, 3760.87) | 790.73 (218.87, 2856.81) | 373.26 (159.29, 874.63) |
| R848 | 125 (98%) | 15276.23 (12541.18, 18607.74) | 16617.64 (12505.27, 22082.37) | 17125.33 (14399.92, 20366.57) |
| LPS | 125 (98%) | 41984.42 (34815.02, 50630.20) | 50284.14 (37237.04, 67902.67) | 38397.59 (33056.99, 44601.00) |
| PGN | 125 (98%) | 68497.11 (58095.45, 80761.12) | 86773.59 (69162.27, 108869.40) | 60138.72 (50395.94, 71765.02) |
| pI:C | 116 (91%) | 1159.34 (705.72, 1904.53) | 686.46 (240.75, 1957.30) | 688.44 (423.00, 1120.45) |
| cGAMP | 110 (86%) | 502.96 (282.19, 896.46) | 528.35 (207.32, 1346.46) | 384.92 (227.13, 652.30) |
| dsRNA | 124 (98%) | 11566.82 (8061.00, 16597.35) | 10832.46 (6569.84, 17860.75) | 5252.92 (3398.31, 8119.69) |
| **IL-1ra** |  |  |  |  |
| RPMI | 84 (66%) | 482.86 (212.17, 1098.88) | 2096.43 (714.31, 6152.81) | 333.43 (146.07, 761.08) |
| Lyovec™ | 89 (70%) | 1097.20 (494.32, 2435.36) | 979.70 (271.33, 3537.44) | 423.21 (182.36, 982.15) |
| R848 | 120 (94%) | 19641.13 (12466.84, 30944.02) | 33795.14 (26899.94, 42457.77) | 16974.26 (9867.01, 29200.91) |
| LPS | 117 (92%) | 9061.53 (4967.03, 16531.27) | 16755.66 (12192.12, 23027.35) | 6792.29 (3813.48, 12097.91) |
| PGN | 114 (90%) | 5049.85 (2673.33, 9539.03) | 8535.96 (5993.65, 12156.63) | 3716.55 (1940.14, 7119.46) |
| pI:C | 117 (92%) | 6710.35 (3963.94, 11359.58) | 7885.76 (3648.16, 17045.62) | 6361.55 (3633.43, 11138.03) |
| cGAMP | 108 (85%) | 3160.15 (1608.65, 6208.01) | 5702.01 (2085.61, 15589.15) | 2403.75 (1191.26, 4850.35) |
| dsRNA | 101 (81%) | 1337.62 (564.09, 3171.91) | 3038.29 (1136.48, 8122.63) | 2125.33 (1042.39, 4333.35) |
| **IL-10** |  |  |  |  |
| RPMI | 49 (39%) | 1.83 (1.36, 2.46) | 1.45 (0.53, 3.93) | 1.88 (1.42, 2.50) |
| Lyovec™ | 93 (73%) | 12.95 (7.86, 21.36) | 11.15 (5.80, 21.45) | 6.94 (4.12, 11.69) |
| R848 | 125 (98%) | 526.78 (455.07, 609.79) | 545.95 (444.97, 669.84) | 508.26 (445.59, 579.73) |
| LPS | 125 (98%) | 312.39 (260.42, 374.73) | 327.24 (246.04, 435.24) | 276.03 (237.94, 320.22) |
| PGN | 125 (98%) | 163.72 (134.91, 198.68) | 155.58 (111.75, 216.61) | 154.81 (129.78, 184.67) |
| pI:C | 88 (69%) | 3.56 (2.43, 5.21) | 6.14 (3.36, 11.22) | 4.77 (3.31, 6.87) |
| cGAMP | 57 (45%) | 2.08 (1.40, 3.09) | 3.17 (1.63, 6.15) | 2.61 (1.97, 3.45) |
| dsRNA | 124 (98%) | 65.87 (49.09, 88.37) | 55.92 (36.68, 85.97) | 45.20 (32.92, 62.07) |

Observations below the detection limit were included as half the value of the lowest detection limit. There were no observations above the detection limit. Abbreviations: KD, Kawasaki disease; TSS, toxic shock syndrome; IFN, interferon; TNF, tumour necrosis factor; IL, interleukin; IL-1ra, receptor antagonist; R848, Resiquimod; LPS, lipopolysaccharide; PGN, peptidoglycan; pI:C, polyinosinic-polycytidylic acid; cGAMP, cyclic guanosine monophosphate-adenosine monophosphate; dsRNA, double stranded RNA.
